# The effects of transitional care intervention on health outcomes in burn patients: a systematic review and meta-analysis

**DOI:** 10.3389/fresc.2026.1743848

**Published:** 2026-02-04

**Authors:** Xiangling Sun, Zhaohong Ding, Kang Yi, Li Zhang, Shuyun Hao, Manli Wu, Lingling Li

**Affiliations:** 1 Department of Nursing, Gansu University of Traditional Chinese Medicine, Lanzhou, Gansu, China; 2 Department of Burn Department I, Gansu Provincial Hospital, Lanzhou, Gansu, China; 3 Department of Nursing, Gansu Provincial Hospital, Lanzhou, Gansu, China; 4 Department of Cardiovascular Surgery, Gansu Provincial Hospital, Lanzhou, Gansu, China

**Keywords:** burn, burns, hospital to home transition, meta-analysis, transitional care

## Abstract

**Background:**

Burn patients often face challenges such as discontinuity of care, high risk of complications, and psychological adaptation difficulties after discharge. Transitional care is a critical measure to ensure their safe transition across different healthcare settings. This study employs a meta-analysis to comprehensively evaluate the impact of transitional care interventions on health outcomes in burn patients, providing evidence to support improved continuity of care.

**Methods:**

EMBASE, the Cochrane Library, PubMed and Web of Science were searched from the establishment of the databases to July 2025. All analysis were conducted using the Revman 5.3.

**Results:**

A total of 4 randomized controlled trials were included in our meta-analysis, encompassing 281 burn patients, with 141 receiving transitional care interventions and 140 receiving usual care. The meta-analysis revealed that transitional care could significantly enhance the quality of life among burned patients [MD = 26.82, 95% CI (4.25, 49.39), *p* = 0.02], and mental health [MD = −16.57, 95% CI (−25.36, −7.78), *p* < 0.001].

**Conclusion:**

Transitional care has been shown to effectively improve patients’ quality of life and improve emotional well-being. Future studies should integrate both subjective reporting and objective assessment metrics to further validate the independent effects and synergistic interactions of distinct intervention modules within transitional care frameworks.

**Systematic Review Registration:**

https://www.crd.york.ac.uk/PROSPERO/ PROSPERO CRD420251042795.

## 1 Introduction

Burn injuries, resulting from exposure to high temperatures, chemicals, radiation, electricity, or friction, impose substantial consequences on both individuals and society (1, 2), and their treatment extends far beyond acute-phase rescue and wound repair. Although modern medical advances have significantly improved survival rates, new challenges have emerged, particularly in reintegrating survivors into their societal roles due to physical and psychosocial complications such as scar contractures, functional impairments, pain, pruritus, and anxiety (3, 4). While high-income countries report relatively low burn-related mortality (5), the high incidence of disability caused by burn scars often leads to visible disfigurement, joint deformities, and loss of function, profoundly reducing quality of life and frequently contributing to anxiety and depression (6, 7). When patients transition abruptly from a highly structured hospital environment with multidisciplinary professional support to a self-management-oriented home and community setting, they often face a critical gap in care. Thus, resolving this crucial “care gap” is a paramount objective for ameliorating the quality of life of patients following treatment.

The concept of “transitional care” emerged in the 1980s and has since evolved into a structured approach aimed at ensuring care continuity across health settings (8, 9). It involves deliberately designed nursing activities that facilitate coordinated and uninterrupted healthcare delivery for patients moving between different levels or institutions of care. As a nursing-led approach, it emphasizes multidisciplinary collaboration, involving a team comprising family doctors, nurses, pharmacists, mental health counselors, social workers, and other specialists (10, 11). Through transitional care services (e.g., home visits, disease management, telephone follow-ups, health education, discharge planning, and community referral coordination), this model ensures continuity of treatment plans and ultimately improves long-term patient outcomes (8, 12). In the field of chronic disease management (e.g., heart failure, stroke), transitional care has been extensively evidenced to effectively reduce readmission rates, enhance quality of life, and decrease healthcare costs (13, 14).

However, research on the application of transitional care in burn patients remains at a preliminary and exploratory stage, with findings characterized by fragmentation and inconsistency. Yang et al. implemented a WeChat-based multidisciplinary continuing rehabilitation management program for patients with deep second- to third-degree hand burns and found that it effectively improved scar conditions and significantly enhanced hand and upper limb function (15). Bayuo et al. conducted an 8-week nurse-led pre-discharge support and proactive WeChat follow-up care intervention, which resulted in improvements in anxiety and depression among burn survivors, though no statistically significant difference was observed in sleep quality (16). In another study, Bayuo et al. reported that the intervention involving pre-discharge support and an 8-week active follow-up nursing program did not yield statistically significant differences in post-burn sexual health outcomes (17). Additionally, studies have indicated that transition programs from hospital to home care help prevent excessive scar complications and improve health-related quality of life in patients with deep partial-thickness burns (18), as well as psychosocial empowerment programs effectively address the psychosocial needs of burn survivors and enhance their early adaptation after discharge (19).

Currently, there is a lack of comprehensive and systematic quantitative integration of existing evidence to identify methodological limitations and evidence gaps in current research, thereby providing direction for future high-quality studies. This study aims to systematically analyze whether transitional care exerts a positive impact on burn survivors by integrating evidence from randomized controlled trials (RCTs).

## 2 Methods

This review followed the guidelines of Preferred Reporting Items for Systematic Reviews and Meta-analyses (PRISMA), and was registered with the International Prospective Register of Systematic Reviews (PROSPERO), and the registration number is CRD420251042795.

### 2.1 Literature search

This Meta-analyses covered four electronic bibliographic databases, including EMBASE (from 1974 to July 2025), the Cochrane Library (from the inception to July 2025), PubMed (from 1950 to July 2025), Web of Science (from 1900 to July 2025).Terms and keywords included those relating to transitional care (e.g., “care transition”, “discharge planning”, “hospital to home”, “hospital discharge”, “continuity of patient care”, “continuing care”, “continuous care”, “extended care”, “aftercare”, “discharge management”, “post-discharge care”, “posthospital care”, “follow-up care”, “continuity of care”, “care coordination”, “integrated care”), and burn (e.g., “burn patient”, “burn reconstruction”, “thermal injury”) were combined with an “AND” term. Search terms were modified according to suggestions from the different databases and are listed in Supplementary Appendix A. Only articles published in English were accepted for language restriction. The original published articles of all references, which including relevant articles and additional articles, suitable for inclusion were retrieved for further analysis.

### 2.2 Inclusion and exclusion criteria

The inclusion criteria: (A) Burned patients; (B) Aged ≥18 years; (C)the study design was RCT.

The exclusion criteria: (A) Duplicate publications; (B) Reviews, letters, expert opinions, case reports, and family-based association studies; (C) Data that was missing, incomplete, or unable to be extracted.

### 2.3 Data extraction and risk of bias

Two reviewers screened independently the information of identified studies (title and abstract) in the research and assessed the full texts of potentially eligible articles to determine whether they met the inclusion criteria. Eligible articles were then extracted data by 2 independent reviewers using a unified table to make a last list of identified studies. If the original data were not reported in the eligible studies, contact the author to obtain data or exclude the study. When there is a disagreement, a third reviewer was consulted.

The risk of bias of the RCTs was independently evaluated by 2 reviewers, the Cochrane Handbook for Systematic Reviews of Interventions Version 5.3.0 of the Cochrane Collaboration was used (20). The following criteria of the Cochrane Handbook was assessed: 1) Random sequence generation, 2) Allocation concealment, 3) Blinding of participants and researchers, 4) Blinding of outcome assessment, 5) Incomplete outcome data, 6) Selective reporting, and 7) Other bias. The results of assessment could be rated as “high risk” (+), “low risk” (−) or “unclear risk” (?). The disagreements were adjudicated by the third reviewers. The methodological quality of the included studies in this meta-analysis was assessed using the 8 criteria from Cuijpers (21), the eight criteria and results are shown in Supplementary Appendix B and C. In general, when no or insufficient information was provided concerning a quality criterion, we rated it as negative.

For each study included in this meta-analysis, the following data were recorded: first author's information and publication year, country, sample size, participants (age and sample size of burn patients in the control and intervention groups), intervening method, and outcome measures (e.g., health-related quality of life, mental health, itch and pain).

### 2.4 Statistical analysis

A quantitative synthesis for the effect size of each study was calculated as the outcomes were continuous data. The mean difference (MD) was calculated to compare the differences between the control and intervention groups. This study adopted random effect model as the main analysis method. I^2^ statistic were used to assess statistical heterogeneity, with I^2^ values of 0%, 25%, 50% and 75% represented no, low, moderate and high heterogeneity, respectively (22). Dimensional analysis was conducted if necessary to explore intervention effects across different dimensions further. The stability of the results was tested by sensitivity analysis. Publication bias were presented by funnel plots. All analysis was conducted using the Review Manager Software (Revman 5.3).

## 3 Results

### 3.1 Search results and study selection

**Figure 1:**
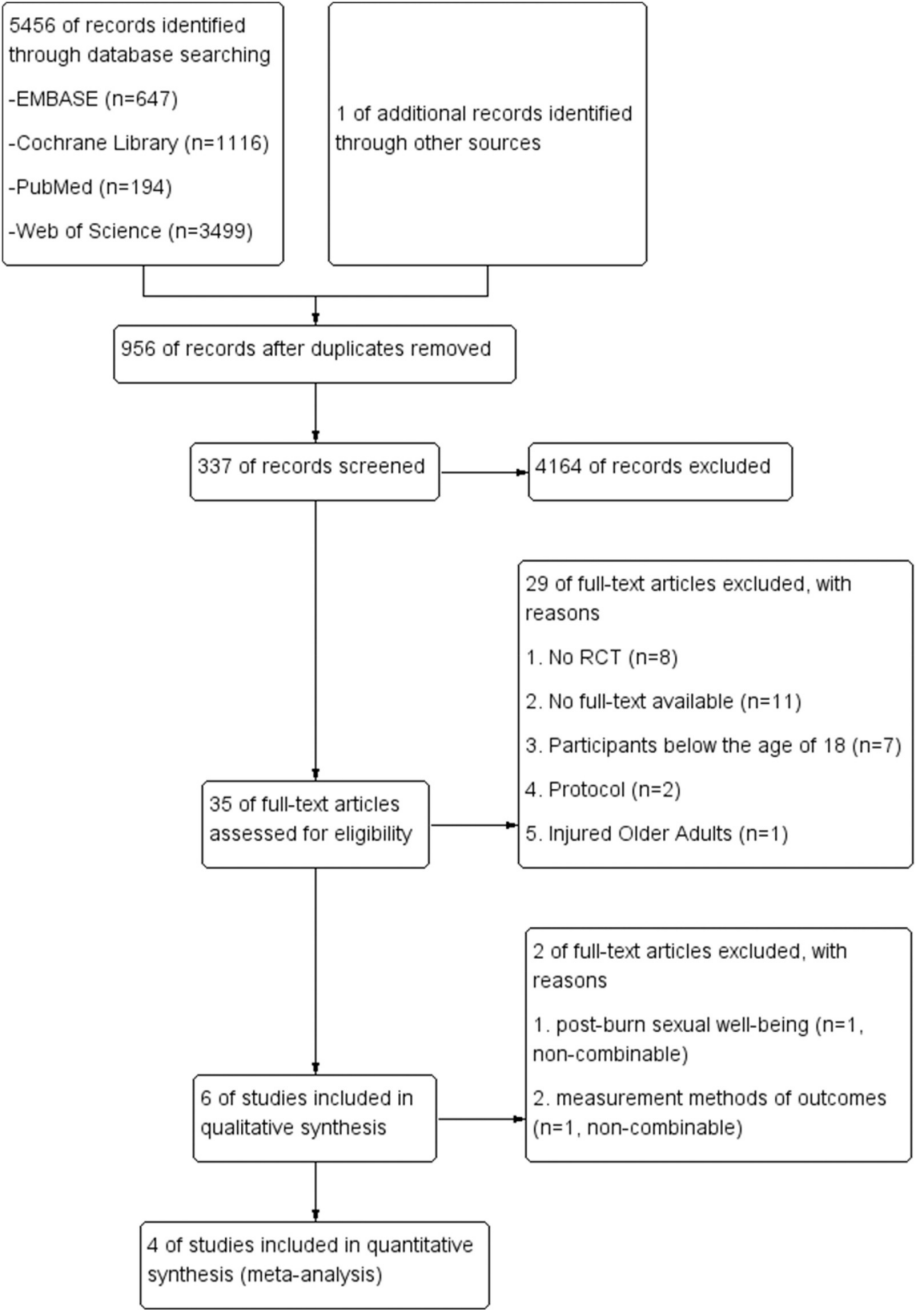
Preferred reporting items for systematic reviews and meta-analyses (PRISMA) flow diagram of the literature search used.

The literature search initially yielded 5,456 records, supplemented by 1 additional reference from manual searches. After removing 956 duplicate entries, we screened the remaining 4,501 records by title and abstract, excluding 4,164 irrelevant studies based on intervention and population criteria. Of the 35 potentially eligible articles retrieved for full-text review, 29 were excluded for not meeting inclusion requirements. After further review of the remaining 6 articles, we found that only 1 paper focused on sexual well-being outcomes (17), and 1 paper employed distinct outcome measurement scales that were non-combinable with other trials' data (19). Ultimately, 4 RCTs qualified for inclusion in our systematic review (23–26). The results of the search progress and study selection are depicted in Figure 1.

### 3.2 Characterization of the included studies

The characteristics of the 4 RCTs are showed in Table 1. This meta-analysis focused on burn survivors with varying severity thresholds: three Iranian studies (23–25) enrolled patients with burns involving >15% total body surface area (TBSA) or dominant hand injuries, while the Australian study (26) included patients with ≤25% TBSA. Health-related data were available for 141 burn patients in the transitional care intervention group and 140 patients in the control group. Participants were predominantly adults (mean age: 34–48 years), stratified into transitional intervention (e.g., home visits, telenursing, mental health screening, rehabilitation programs) and control groups.

**Table 1:** Characteristics of included studies.

Author, year	Country	Sample Size	Participants	Interventions	Outcome measures
Heydarikhayat et al., 2018 (23)	Iran	86	—Burn severity >15% —Con = 44 age = 35.02 ± 13.1 —Int = 42 age = 38.42 ± 14.0	Home visits, telenursing, and referral to specialists or health education centers	BSHS-B, GHQ-28, BPI, VSS, and VAS
Heydarikhayat et al., 2023 (24)	Iran	90	—Burn severity >15% —Con = 45 age = 34.46 ± 12.8 —Int = 45 age = 39.13 ± 14.0	Home care plan that included mental health screening, education, and referral to specialists and follow-ups.	GHQ-28
Seyedoshohadaee et al., 2022 (25)	Iran	60	—The burn of the dominant hand —Con = 30 age = 36.53 ± 14.7 —Int = 30 age = 34.1 ± 13.2	A 5-week nursing rehabilitation program, including regular health instructions, health education, psychological counseling sessions, hand rehabilitation movements, and so on.	BSHS-B
Plaza et al., 2023 (26)	Australia	45	—Burn severity ≤ 25% —Con = 22 age = 45.1 ± 16.0 —Int = 23 age = 48.4 ± 14.2	a 6-week exercise program delivered by home-based telerehabilitation	BSHS-B, VAS

Con, control group; Int, intervention group; BSHS-B, the Burns Specific Health Scale Brief; GHQ-28, General Health Questionnaire-28; BPI, Brief Pain Inventory; VSS, the Vancouver Scar Scale; VAS, the Visual Analogue Scale.

Interventions were heterogeneous, spanning home-based care (e.g., health education, psychological support), telenursing, and structured rehabilitation programs (5–6 weeks). Outcome measures included the Burns Specific Health Scale-Brief (BSHS-B), General Health Questionnaire-28 (GHQ-28), pain/scar scales (BPI, VSS), and Visual Analog Scale (VAS). Key limitations included small sample sizes and regional concentration (3/4 studies from Iran), potentially affecting generalizability. However, consistent use of validated tools (e.g., BSHS-B) enhanced outcome comparability.

### 3.3 Quality assessment

**Figure 2:**
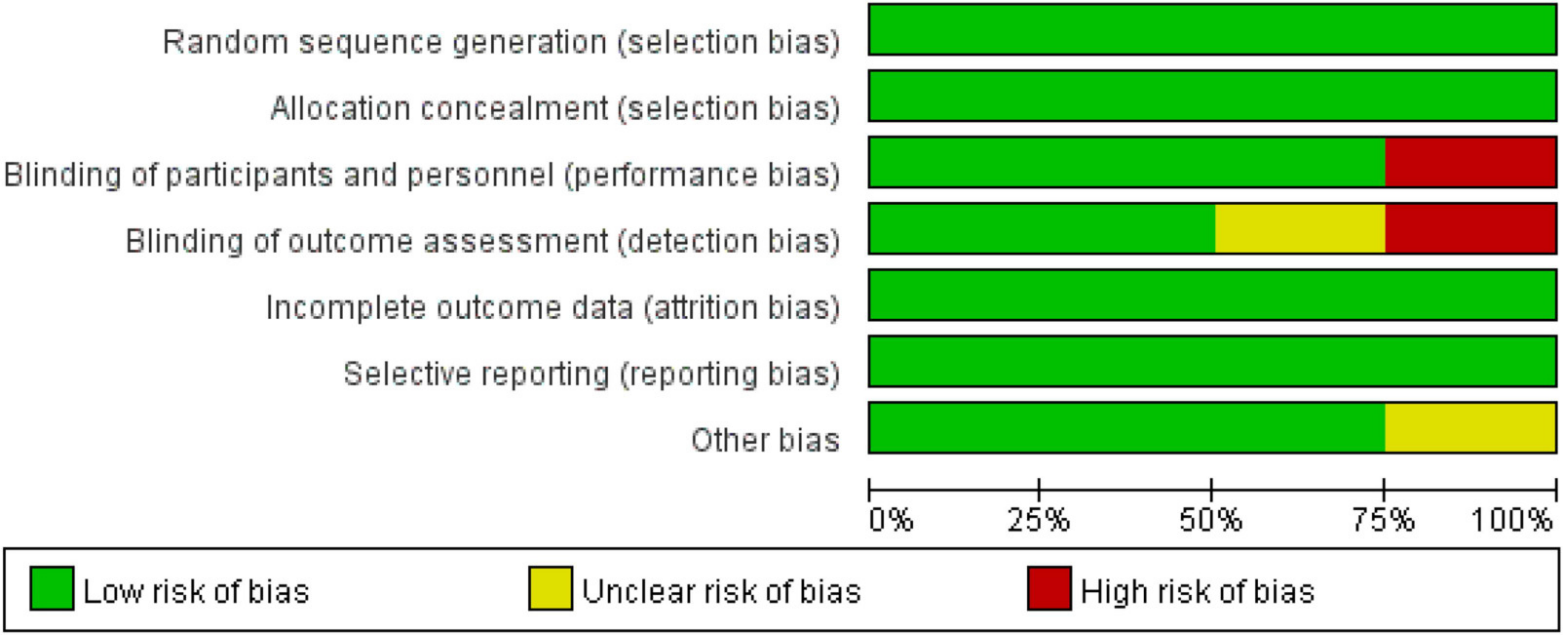
Risk of bias graph.

**Figure 3:**
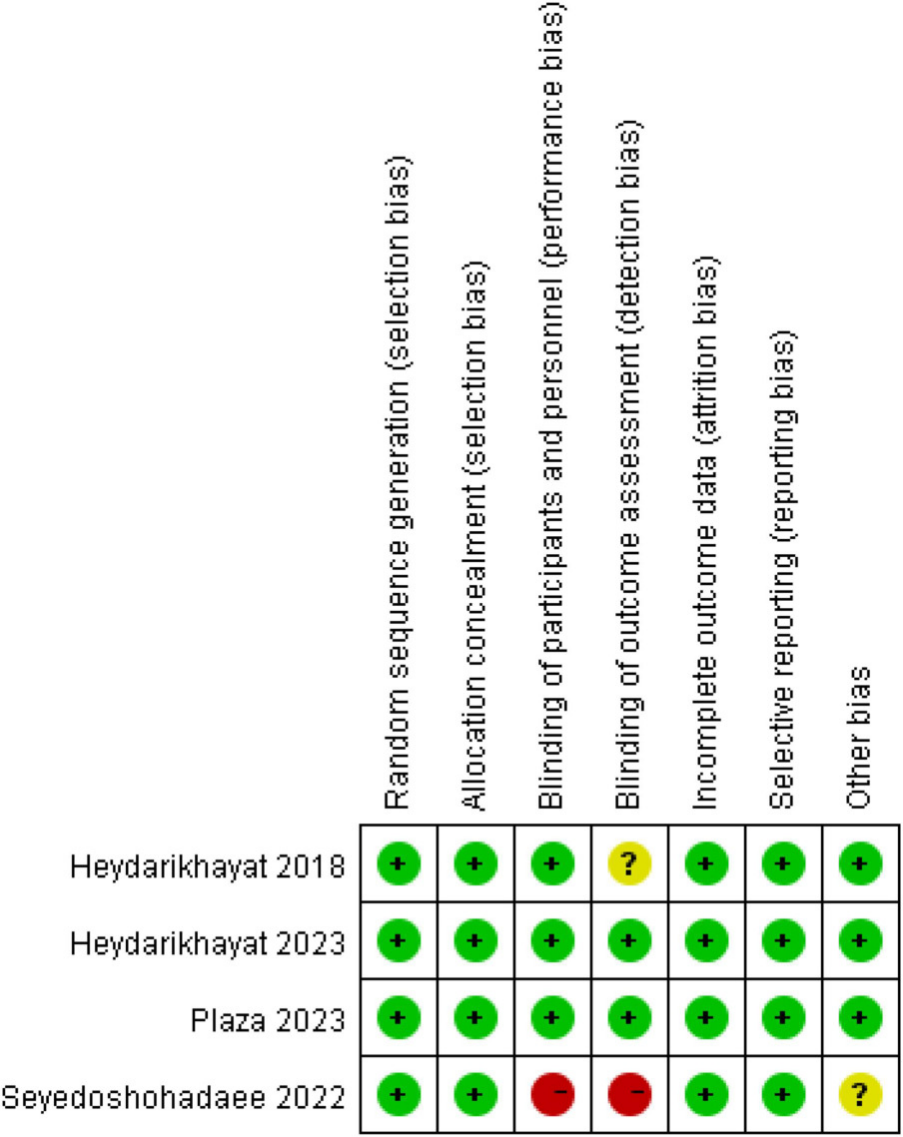
Risk of bias summary.

Risk of bias are shown for all included studies in Figure 2 and for each study in Figure 3. In general, the four trials demonstrated low risk of bias in random sequence generation, allocation methods, complete outcome reporting, and selective reporting. All four trials provided detailed descriptions of randomization methods, including the use of PASS software to generate randomized lists (*n* = 3) and random number tables (*n* = 1), and ensured participant comparability through block randomization. Three trials implemented blinding of investigators and participants, while two trials blinded the outcome measurement. One trial reported no participant dropout, whereas the remaining three trials documented attrition reasons, including high treatment costs, preference for herbal and home therapies, relocation, or partial recovery.

### 3.4 Effects of transitional care intervention on burned patient

#### 3.4.1 Health-related quality of life

**Figure 4:**
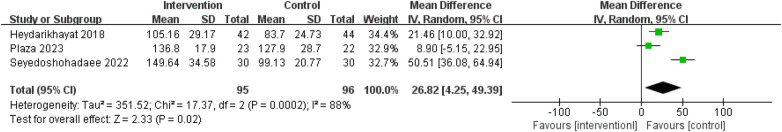
Effects of transitional care intervention on health-related quality of life.

A total of 3 RCTs assessing health-related quality of life in burn patients using the Burn Specific Health Scale-Brief (BSHS-B) were included (23, 25, 26), involving 95 participants in the intervention group and 96 in the control group (Figure 4). Meta-analysis revealed substantial heterogeneity in health-related quality of life outcomes across studies (I² = 88%, *p* < 0.001). Transitional care significantly improved health-related quality of life [MD = 26.82, 95% CI (4.25, 49.39), *p* = 0.02]. Sensitivity analysis showed that the results were stable and reliable.

#### 3.4.2 Mental health

**Figure 5:**
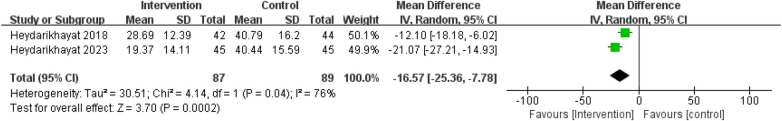
Effects of transitional care intervention on mental health.

**Figure 6:**
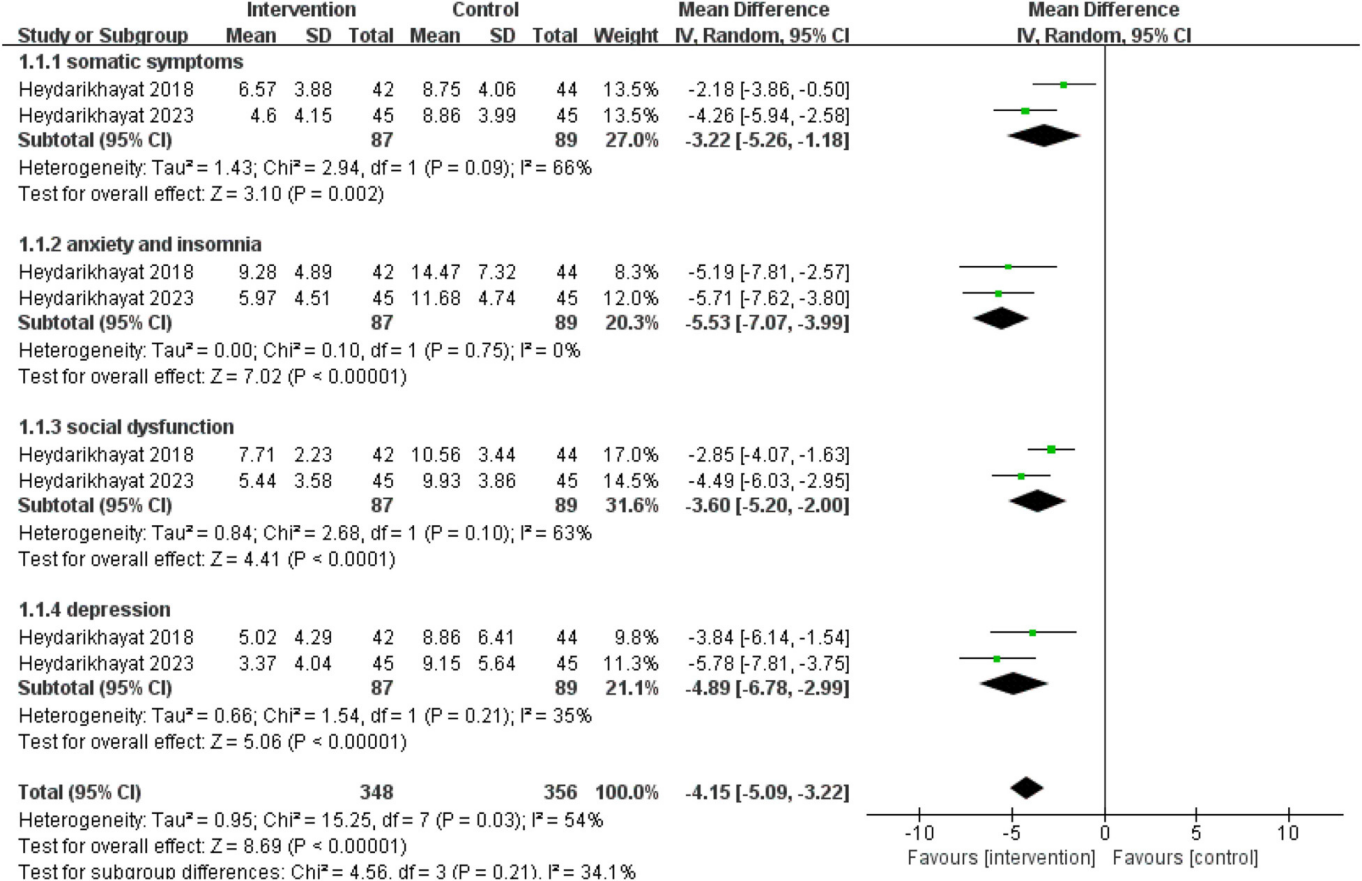
Effects of transitional care intervention on domains of mental health.

A total of 2 RCTs utilizing the 28-item General Health Questionnaire (GHQ-28) for mental health assessment were analyzed, involving 87 participants in intervention group and 89 participants in control group (23, 24). The meta-analysis showed that transitional care reduced psychological distress scores in burn patients [MD = −16.57, 95% CI (−25.36, −7.78), *p* < 0.001] (Figure 5). Dimensional analysis was performed to examine the intervention effects across different dimensions of GHQ-28 (Figure 6). The results demonstrated that transitional care significantly improved burn patients' physical symptoms [MD = −3.22, 95% CI (−5.26, −1.18), *p* = 0.002], anxiety and insomnia [MD = −5.53, 95% CI (−7.07, −3.99), *p* < 0.001], social function [MD = −3.60, 95% CI (−5.20, −2.00), *p* < 0.001], and depression [MD = −4.89, 95% CI (−6.78, −2.99), *p* < 0.001]. Sensitivity analysis showed that the results were stable and reliable.

#### 3.4.3 Itch and pain

**Figure 7:**
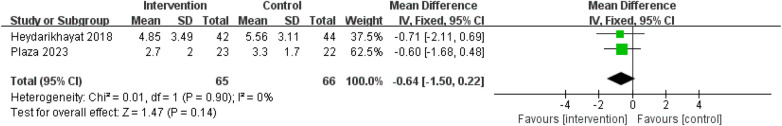
Effects of transitional care intervention on itch and pain.

Itch and pain were evaluated in 2 trials, there were 65 participants in the intervention group and 66 participants in the control group (Figure 7) (23, 26). There was no heterogeneity in executive function among studies (I^2^ = 0%, *p* = 0.90). Meta-analysis showed that transitional care did not significantly improve itch and pain in burn patients [MD = −0.64, 95% CI (−1.50, 0.22), *p* = 0.14]. Sensitivity analysis showed that the results were stable and reliable.

## 4 Discussion

By synthesizing evidence from four RCTs published between 2018 and 2023, this meta-analysis evaluated the effects of transitional care on multiple patient-centered outcomes, including health-related quality of life, mental health, as well as itch and pain. The findings highlight the beneficial impact of transitional care on both mental health and overall quality of life in burn survivors, and provide empirical evidence for transitional care in burn rehabilitation, which has long lacked high-quality evidence. However, current evidence did not demonstrate a significant effect on itch or pain symptoms.

### 4.1 Interpretation of results and comparison with previous research

To our knowledge, this meta-analysis represents the first systematic investigation into the association between transitional care and outcomes in burn survivors. Michael et al. (27) evaluated the effectiveness of nurse-led transitional care on quality of life, mortality, and readmission among adult stroke survivors. Among the 18 included trials, 16 reported a moderate yet significant effect of nurse-led transitional care programs on quality of life. It is worth noting that the review incorporated eight distinct instruments with varied psychometric properties to assess quality of life, thereby capturing the benefits of transitional care interventions across multiple dimensions in stroke survivors. The present meta-analysis corroborates the findings from Campbell et al.'s study, which utilized the BSHS-B to evaluate quality of life in burn survivors and likewise demonstrated a positive effect of transitional care on health-related quality of life. Transitional care improves the quality of life for burn patients not through a single pathway, but rather through a multidimensional and interconnected mechanism of action: Firstly, through platforms such as WeChat, multidisciplinary teams (including rehabilitation therapists and nurses) provide continuous, specialized, and personalized guidance that enhances patients' knowledge and self-management skills, enabling them to better adhere to demanding rehabilitation regimens (28). Secondly, healthcare professionals can remotely monitor the recovery progress that patients report in real-time on the platform (such as sending photos of scars or describing pain levels), enabling timely detection of issues, immediate corrective guidance, and providing patients with a continuous sense of security, thereby alleviating their anxiety (26, 29). Thirdly, multidisciplinary team collaboration provides patients with a unified and coherent rehabilitation plan, which reduces the burden on patients and their families of navigating and coordinating between multiple departments, enhances the overall care experience, and thereby indirectly improves their quality of life (30). Lastly, providing strong emotional support, implementing cognitive-behavioral interventions, and facilitating peer sharing and mutual encouragement can reduce the impact of post-burn psychological trauma and social anxiety on quality of life, while strengthening confidence in reintegrating into society (31). Therefore, future studies should develop more concise and effective transitional care interventions based on the above mechanisms, aiming to improve patients' quality of life while reducing the workload of healthcare providers.

A meta-analysis by Zou et al. suggested that transitional care significantly improves both physical and mental health outcomes in older adults with chronic diseases compared to conventional care (32). They found that transitional care led to notable short- and long-term enhancements in psychological well-being, physical function, and vitality after discharge—a trend consistent with the observations in the current study. Burn patients face substantial emotional and psychological challenges, with high rates of anxiety, depressive symptoms, post-traumatic stress disorder, and difficulties in social reintegration and occupational development (33). Therefore, early identification of high-risk individuals and the provision of multidimensional support (e.g., specialized medical interventions, active rehabilitation strategies, and innovative psychological assistance methods) are essential for burn patients.

### 4.2 Implications for clinicians

This study aligns with findings from research on stroke and chronic disease patients, indicating that transitional care offers universal benefits for individuals with complex rehabilitation needs and should be regarded as an essential component rather than an ancillary service within the standard rehabilitation pathway for such patients. Transitional care should be implemented through the synergistic integration of multidisciplinary teams, structured care pathways, and digital tools (34). During its execution, emphasis must be placed on risk screening, stratified intervention, and personalized management (35). For instance, patients with poor self-management skills may benefit from intensified platform guidance and feedback, while those at high psychological risk should receive early integration of cognitive-behavioral interventions and peer support (36). This approach ultimately facilitates a shift from a hospital-centered to a patient-centered continuum of care.

### 4.3 Implications for future research

Research on the impact of transitional care interventions on health outcomes in burn patients remains limited, and existing studies often broadly encompass all intervention elements without distinction. Future investigations into transitional care for burn patients should focus on deconstructing this complex “black box” of intervention by examining the independent and synergistic contributions of its various components, such as remote monitoring, professional guidance, and peer support. By integrating both subjective and objective metrics, studies should explore the optimal timing for initiating interventions and ideal duration to determine the most cost-effective protocols.

In addition, there is a need to develop and validate a multidimensional tool for assessing core outcomes in burn populations, which would improve comparability across studies. Further research should identify barriers and facilitators to implementing transitional care in different resource settings (37), and explore practical strategies for enabling information exchange among hospitals, community health centers, and rehabilitation facilities to ensure seamless post-discharge care (38). Finally, greater emphasis must be placed on personalizing interventions to identify the most beneficial approaches for different patient subgroups based on burn characteristics, psychological risk, and social support levels (39). This will ultimately provide a robust evidence base for developing standardized, precise, and evidence-based transitional rehabilitation strategies.

### 4.4 Strengths and limitations

There are several advantages in this study. This meta-analysis is the first systematic review to evaluate the effect of transitional care on multiple health outcomes in burn patients, and found that transitional care can improve health-related quality of life and mental health in burn survivors. Second, only RCTs in English were included in this meta-analysis. Nevertheless, this study has some limitations. Firstly, restricted by the scope of the search (which did not cover study registries and grey literature) and the limited number of included studies (only four), the sample size for analysis is relatively small, which contributes to uncertain results and potentially inaccurate risk estimates. Secondly, we assessed quality of life using only one scale, the BSHS, which may not comprehensively capture all aspects relevant to patients' quality of life. For these limitations, our results should be carefully interpreted.

## 5 Conclusion

The transitional care intervention shows promise in enhancing the quality of life and mental health among burn patients after discharge, with a potentially greater improvement trend compared to routine care. There was a limited sample size in the included studies, thus the results of the present study need to be cautiously interpreted. Future studies should aim to disentangle the independent and synergistic contributions of specific intervention components, incorporate both subjective and objective outcome measures to comprehensively evaluate effectiveness, and investigate pathways for enabling information exchange between hospitals and community health centers to support continuous care.

## Data Availability

The original contributions presented in the study are included in the article/Supplementary Material, further inquiries can be directed to the corresponding author.
