# Supplementary material for: The effects of transitional care intervention on health outcomes in burn patients: a systematic review and meta-analysis
Source: Front Rehabil Sci. 2026 Feb 4;7:1743848. doi: 10.3389/fresc.2026.1743848 (PMC12913459; doi:10.3389/fresc.2026.1743848)

**Appendix A.** Full search terms

Table A.1 Embase search terms.

| Embase search terms | |
| --- | --- |
| #1 | 'transitional care'/exp |
| #2 | 'transitional care':ab,ti OR 'care transition':ab,ti OR 'discharge planning':ab,ti OR 'hospital to home':ab,ti OR 'hospital discharge':ab,ti OR 'continuity of patient care':ab,ti OR 'continuing care':ab,ti OR 'continuous care':ab,ti OR 'extended care':ab,ti OR 'aftercare':ab,ti OR 'discharge management':ab,ti OR 'post-discharge care':ab,ti OR 'posthospital care':ab,ti OR 'follow-up care':ab,ti OR 'continuity of care':ab,ti OR 'care coordination':ab,ti OR 'integrated care':ab,ti |
| #3 | #1 OR #2 |
| #4 | 'burn'/exp |
| #5 | 'burn patient':ab,ti OR 'burn reconstruction':ab,ti OR 'thermal injury':ab,ti |
| #6 | #4 OR #5 |
| #7 | #3 AND #6 |

Table A.2 Cochrane search terms.

| Cochrane search terms | |
| --- | --- |
| #1 | MeSH descriptor: [Transitional Care] explode all trees |
| #2 | (transitional care):ti,ab,kw OR (care transition):ti,ab,kw OR (discharge planning):ti,ab,kw OR (hospital to home):ti,ab,kw OR (hospital discharge):ti,ab,kw |
| #3 | (continuity of patient care):ti,ab,kw OR (continuing care):ti,ab,kw OR (continuous care):ti,ab,kw OR (extended care):ti,ab,kw OR (aftercare):ti,ab,kw |
| #4 | (discharge management):ti,ab,kw OR (post-discharge care):ti,ab,kw OR (posthospital care):ti,ab,kw OR (follow-up care):ti,ab,kw OR (continuity of care):ti,ab,kw |
| #5 | (care coordination):ti,ab,kw OR (integrated care):ti,ab,kw |
| #6 | #1 OR #2 OR #3 OR #4 OR #5 |
| #7 | MeSH descriptor: [Burns] explode all trees |
| #8 | (burn patient):ti,ab,kw OR (burn reconstruction):ti,ab,kw OR (thermal injury):ti,ab,kw |
| #9 | #7 OR #8 |
| #10 | #6 AND #9 |

Table A.3 Pubmed search terms.

| Pubmed search terms | |
| --- | --- |
| #1 | "Transitional Care"[Mesh] OR "transitional care"[tiab] OR "care transition"[tiab] OR "discharge planning"[tiab] OR "hospital to home"[tiab] OR "patient discharge"[tiab] OR "continuity of patient care"[tiab] OR "continuing care"[tiab] OR "continuous care"[tiab] OR "extended care"[tiab] OR "aftercare"[tiab] OR "discharge management"[tiab] OR "post-discharge care"[tiab] OR "posthospital care"[tiab] OR "follow-up care"[tiab] OR "continuity of care"[tiab] OR "care coordination"[tiab] OR "integrated care"[tiab] |
| #2 | "Burns"[Mesh] OR "burn"[tiab] OR "burn injury"[tiab] OR "thermal injury"[tiab] |
| #3 | #1 AND #2 |

Table A.4 Web of Science search terms.

| Web of Science Core Collection search terms | |
| --- | --- |
| #1 | transitional care (Title) OR care transition (Abstract) OR discharge planning (Abstract) OR hospital to home (Abstract) OR hospital discharge (Abstract) OR continuity of patient care (Abstract) OR continuing care (Abstract) OR continuous care (Abstract) OR extended care (Abstract) OR aftercare (Abstract) OR discharge management (Abstract) OR post-discharge care (Abstract) OR posthospital care (Abstract) OR follow-up care (Abstract) OR continuity of care (Abstract) OR care coordination (Abstract) OR integrated care (Abstract) |
| #2 | burn (Title) or burns (Abstract) or burn reconstruction (Abstract) or thermal injury (Abstract) or burn patient (Abstract) |
| #3 | #1 AND #2 |

**Appendix B.** Risk of bias summary.


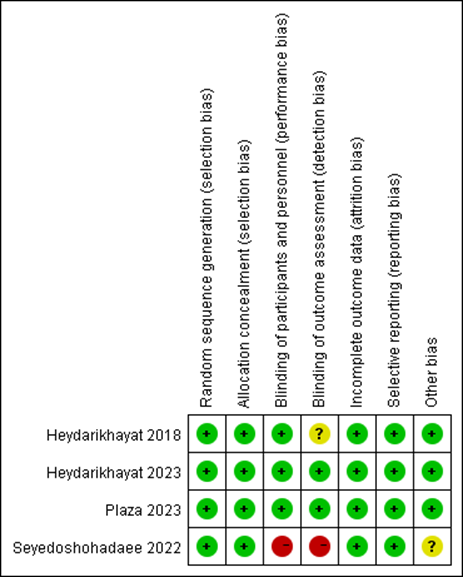


Appendix C**. Effects of transitional care intervention on mental health.**


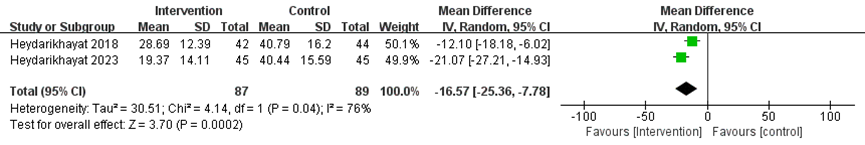


Appendix D. **Effects of transitional care intervention on itch and pain.**


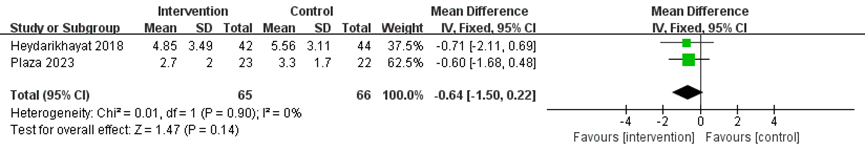

Supplement: Supplementary file 1 [file Datasheet1.docx]
